# Long Head Biceps Tendon Angle Shows the Highest Sensitivity and Long Head Biceps Tendon-Groove Distance the Highest Specificity for the Diagnosis of Biceps Pulley Lesions Using Conventional Magnetic Resonance Imaging

**DOI:** 10.1016/j.asmr.2025.101253

**Published:** 2025-08-22

**Authors:** Javier Ardebol, Roger Erosa-Villarreal, Edwin Valencia-Ramón, Alejandro López-Villers, Guillermo Franco-del Río, Patrick J. Denard, Frank Martetschläger, Juan Cosme-Labarthe, Alexandre Lädermann, Juan Pablo Muñoz, Alberto Guevara-Alvarez

**Affiliations:** a Instituto de Hombro IDH, Hospital Angeles Querétaro, Querétaro, México; b Instituto Queretano de Alta Especialidad en Ortopedia (IQAEO). Hospital Ángeles de Querétaro, Qro, México; c Universidad Anáhuac Querétaro, México; d Oregon Shoulder Institute, Medford, Oregon, U.S.A.; e Deutsches Schulterzentrum, ATOS Klinik, Munich, Germany; f Department of Radiology, Instituto Nacional de Ciencias Médicas y Nutrición Salvador Zubirán, Mexico City, Mexico; g Division of Orthopaedics and Trauma Surgery, Department of Surgery, Faculty of Medicine, University of Geneva, Geneva, Switzerland; h Departamento de Radiología, Sistema Musculoesquelético, Clínica MEDS, Santiago, Chile

## Abstract

**Purpose:**

To determine the diagnostic performance and inter-rater agreement for magnetic resonance imaging (MRI) signs of long head biceps tendon (LHBT) instability and overall, using conventional MRI for the diagnosis of biceps pulley lesions.

**Methods:**

In this retrospective analysis, conventional MRIs were reviewed by 5 assessors for the presence or absence of biceps pulley lesions and 6 specific MRI signs. Diagnostic performance of pulley lesion and sign detection using MRI was tested using arthroscopy as the reference standard. Interobserver agreement was measured with Kappa statistics and diagnostic performance with sensitivity, specificity, negative and positive predictive values overall and for radiologists and surgeons.

**Results:**

A total of 60 MRIs, 30 with biceps pully lesions and 30 without, were included. Overall, diagnostic performance metrics for MRI included a sensitivity of 81%, specificity of 79%, positive predictive value of 80%, and negative predictive value of 80%. Interobserver reliability analysis revealed moderate agreement overall, with a global kappa value of 0.59. LHBT angle showed the highest sensitivity (84%) and LHBT-groove distance showed the greatest specificity (98%). Both radiologists and surgeons reported similar diagnostic accuracy through MRI.

**Conclusions:**

Overall, conventional MRI had an acceptable diagnostic performance, with sensitivity, specificity, and predictive values of approximately 80%. Among the evaluated signs, the LHBT angle had the highest sensitivity, whereas the LHBT-groove distance showed the greatest specificity. Interobserver reliability was moderate overall, though some observer pairs achieved substantial agreement. However, there was variability across diagnostic signs.

**Level of Evidence:**

Level III, retrospective comparative study.

The biceps pulley serves as a major soft-tissue stabilizer of the long head biceps tendon (LHBT), preventing its medial and inferior dislocation.^1^, ^2^, ^3^ Structurally, the pulley system is formed primarily by the superior glenohumeral ligament and the coracohumeral ligament at the apex of the rotator interval.^3^, ^4^, ^5^ Acute trauma, repetitive microtrauma, or degenerative changes can compromise these structures, leading to biceps tendon instability, impaired shoulder function, and anterior shoulder pain.^3^^,^^4^ Pulley lesions, with an arthroscopically confirmed prevalence of approximately 7%, are not uncommon; however, clinical evaluation remains challenging because of equivocal physical examination findings, such as pain with palpation of the bicipital groove, and pain with resisted elbow flexion (i.e., Speed’s test).^6^ Imaging often is required to aid diagnosis, with conventional magnetic resonance imaging (MRI) serving as one of the options.^3^^,^^7^

Although magnetic resonance (MR) arthrography has shown excellent accuracy in detecting pulley lesions, few studies have evaluated the performance of conventional MRI—a modality widely used in the assessment of shoulder pathology.^3^^,^^7^, ^8^, ^9^ Existing research is limited, with results varying significantly. In a previous analysis of 86 conventional MRI scans, Zappia et al.^10^ examined the diagnostic performance and inter-rater reliability of 7 MRI signs of LHBT instability (i.e., chondral print, humeral head subchondral bone edema at the chondral print, LHBT angle, LHBT-groove distance, LHBT subluxation or dislocation on the axial plane, detour and displacement sign); however, these findings have not been broadly validated.

The purposes of this study were to determine the diagnostic performance and inter-rater agreement for MRI signs of LHBT instability and to evaluate the use of conventional MRI for the diagnosis of biceps pulley lesions. We hypothesized that the displacement sign, LHBT-groove distance, and LHBT angle would provide the greatest diagnostic value.

## Methods

### Study Design

A retrospective case-control review was conducted on prospectively collected data on adult patients who underwent shoulder arthroscopy between 2019 and 2022 at a single institution. Inclusion criteria included a preoperative noncontrast MRI with a 1.5-Tesla magnet and subsequent arthroscopic evaluation. Patients younger than 18 years of age were excluded, as well as those with an MRI with motion artifact, history of ipsilateral shoulder surgery, proximal humerus or glenoid fracture, signs of multidirectional shoulder instability, or incomplete or inadequate operative documentation. Patients with an arthroscopically confirmed pulley lesion comprised the study group, whereas those with an arthroscopically intact biceps pulley were categorized as the control group. Arthroscopic findings were considered the gold standard. The protocol was approved by the local institutional review board before the study’s inception. The requirement for informed consent was waived.

### Arthroscopy

**Fig 1:**
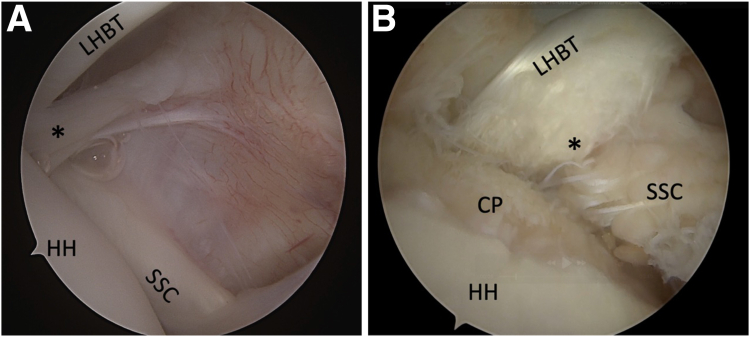
Arthroscopic posterior portal view of a left shoulder showing the integrity of the medial bicipital pulley (∗) in relation to the LHBT and SSC tendon (A) and discontinuity of the medial pulley (∗) associated with an upper third SSC tear (B). (CP, chondral print; HH, humeral head; LHBT, long head biceps tendon; SSC, subscapularis tendon.)

**Fig 2:**
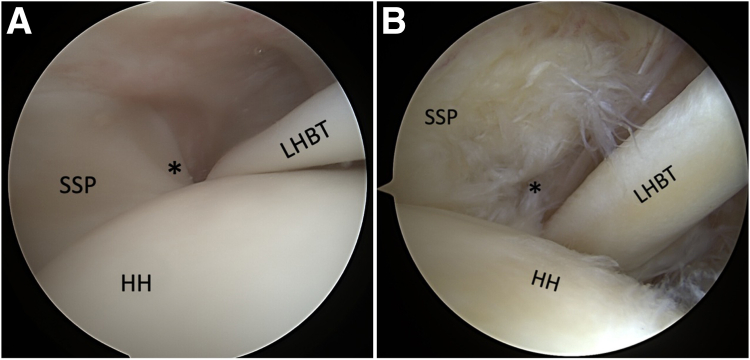
Arthroscopic posterior portal view of a left shoulder showing the integrity of the lateral bicipital pulley (∗) in relation to the LHBT and SSP tendon (A) and discontinuity of the lateral pulley (∗) associated with an anterior SSP tear (B). (HH, humeral head; LHBT, long head biceps tendon; SSC, subscapularis tendon.)

All arthroscopic procedures were performed by a fellowship-trained shoulder surgeon (A.G.). At the time, the surgeon was aware of the MRI interpretation before surgery. Intraoperatively, patients were positioned in the lateral decubitus position, and conventional portals were used (i.e., posterior, anterosuperior and lateral). Subscapularis tendon integrity, the LHBT complex, and surrounding structures were evaluated with 30° and 70° arthroscopes inserted through the posterior portal. The presence of biceps medial pulley lesion (Fig 1), lateral pulley lesion (Fig 2), signs of tendinopathy of the LHBT, and the integrity of rotator cuff muscles adjacent to the rotator interval were documented. Therapeutic procedures performed at arthroscopy were documented.

### Preoperative Imaging: Acquisition and Interpretation

Each patient underwent conventional shoulder MRI on a 1.5-Tesla whole-body scanner equipped with a 16-channel shoulder coil at varying institutions (i.e., outside MRIs). The shoulder was positioned neutrally in accordance with a standardized protocol. Imaging sequences included triplanar intermediate-weighted turbo-spin echo sequences (TSE) with spectral fat suppression, a sagittal T2-weighted TSE sequence, and a coronal T1-weighted TSE sequence. No intra-articular or intravenous contrast was administered for any examination.

**Fig 3:**
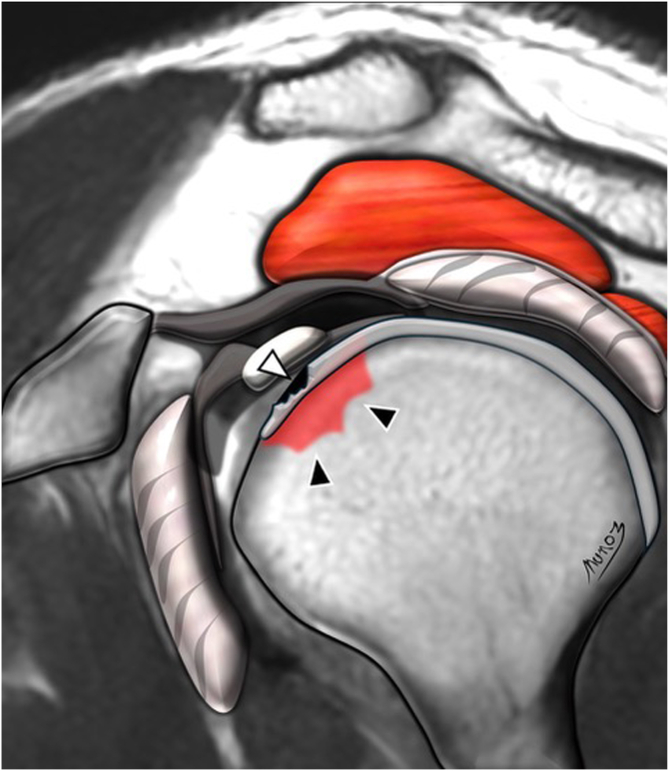
Chondral print sign (white arrowhead) and humeral head subchondral bone edema (black arrowheads) are shown in a sagittal view of a T1-weighted right shoulder MRI. (MRI, magnetic resonance imaging.)

**Fig 4:**
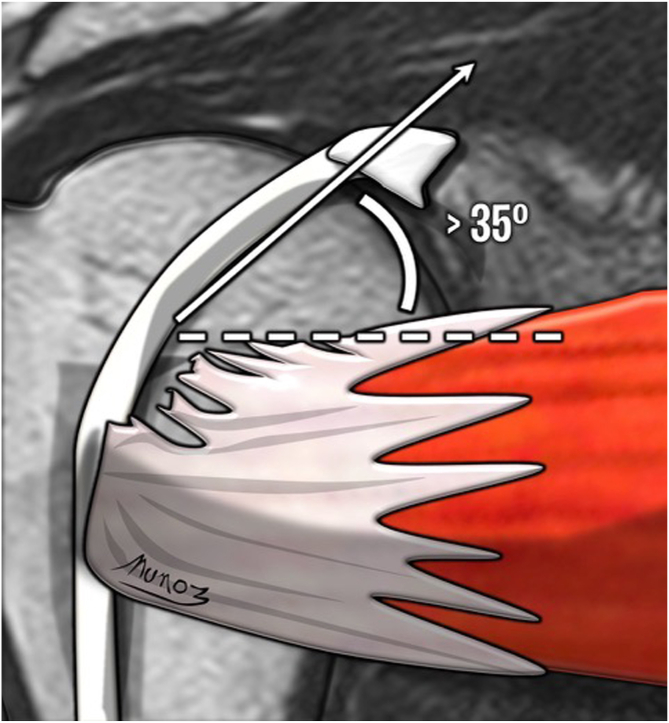
LHBT angle sign is shown in a coronal view of a T1-weighted right shoulder MRI. The angle is measured between the intra-articular portion of the LHBT and the superior edge of the SSC tendon. An angle >35° is considered a positive sign. (LHBT, long head biceps tendon; MRI, magnetic resonance imaging; SSC, subscapularis tendon.)

**Fig 5:**
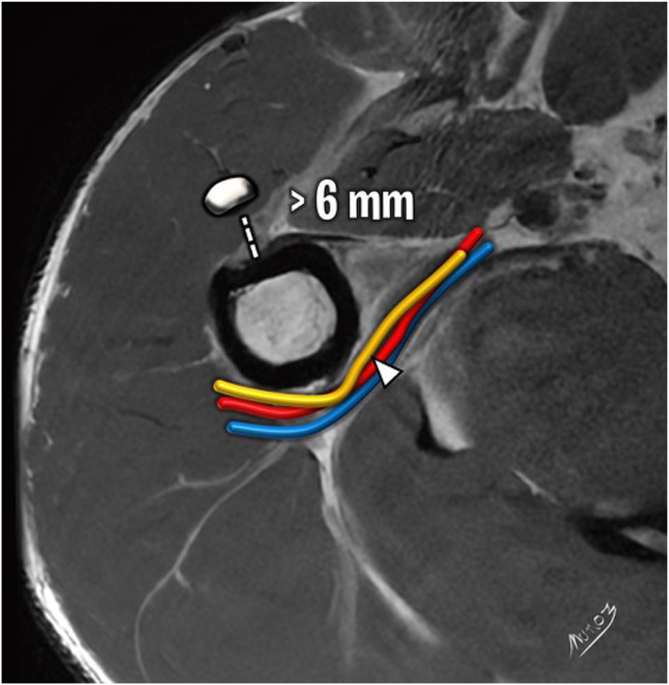
LHBT-groove distance sign (dashed line) is shown in an axial view of a T1-weighted right shoulder MRI. The axillary nerve is indicated with a white arrowhead. The lines represent the axillary neurovascular bundle comprised of the axillary artery (red line), vein (blue line), and nerve (yellow line). (LHBT, long head biceps tendon; MRI, magnetic resonance imaging.)

**Fig 6:**
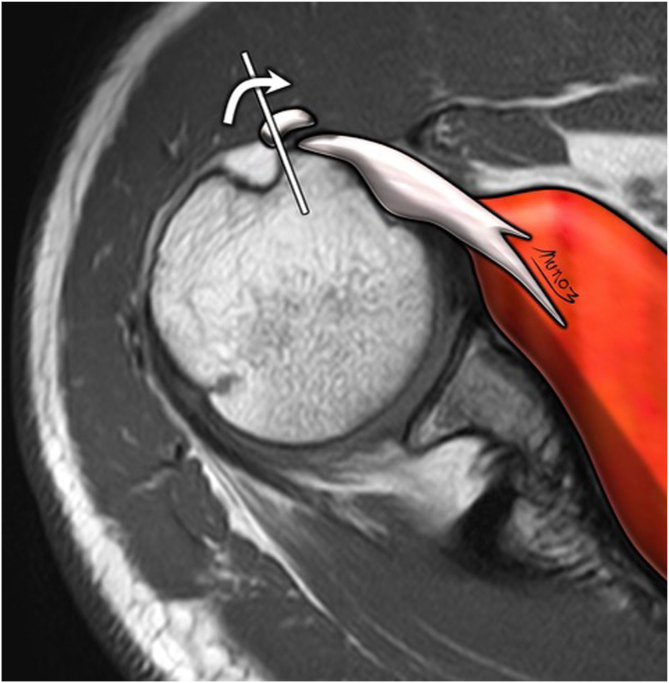
LHBT subluxation sign is shown in an axial view of a T1-weighted right shoulder MRI. The white line marks the limit of the bicipital groove. The white curved arrow shows the direction of a subluxed or displaced LHBT from the bicipital groove. (LHBT, long head biceps tendon; MRI, magnetic resonance imaging.)

**Fig 7:**
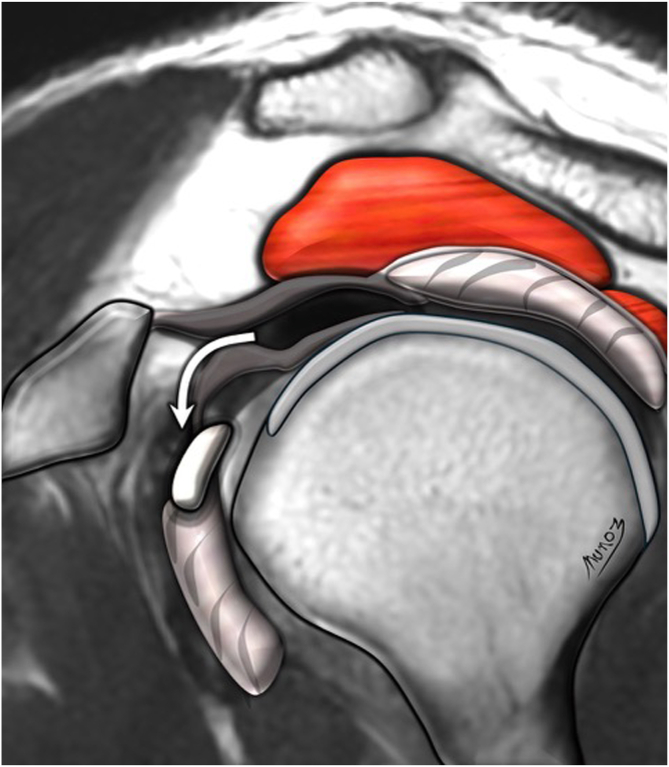
Displacement sign (white curved arrow) is shown in a sagittal view of a T1-weighted right shoulder MRI. (MRI, magnetic resonance imaging.)

Five independent assessors—comprising 3 fellowship-trained shoulder surgeons (assessors 1, 2, and 3) and 2 musculoskeletal radiologists with at least 10 years of experience in shoulder imaging (assessors 4 and 5)—evaluated the MRIs. All assessors were blinded to clinical and surgical information and analyzed the scans using predefined MRI assessment criteria. Specifically, they assessed 6 of the 7 MRI signs of LHBT instability described by Zappia et al.^10^: chondral print (Fig 3), humeral head subchondral bone edema at the chondral print, LHBT angle (Fig 4), LHBT-groove distance (Fig 5), LHBT subluxation or dislocation on the axial plane (Fig 6), and displacement sign (Fig 7). The detour sign was excluded because of its poor diagnostic performance in previous studies. Each assessor recorded the presence or absence of the individual signs and ultimately determined whether a biceps pulley lesion was present.

### Statistical Analysis

With arthroscopy as the gold standard, the sensitivity, specificity, positive predictive value (PPV), negative predictive value (NPV), and accuracy of each sign, as reported by each assessor in the identification biceps instability, were calculated from merged interpretations from all assessors, and compared between surgeons (assessors 1, 2, and 3) and radiologists (assessors 4 and 5). If at least 1 of the 6 signs was present, the MRI was considered positive. Cohen’s kappa (k) was used to evaluate inter-rater agreement for each sign. Cohen's kappa coefficient is interpreted as follows: a value of ≤0 indicates no agreement, 0.01 to 0.20 indicates slight agreement, 0.21 to 0.40 indicates fair agreement, 0.41 to 0.60 indicates moderate agreement, 0.61 to 0.80 indicates substantial agreement, and 0.81 to 1.00 indicates a nearly perfect agreement, as classified by Landis and Koch.^11^ To compare the diagnostic accuracy between assessors and radiologists versus surgeons, the area under the receiver operating curve was used. A threshold of 0.05 was used to denote statistical significance. The data were evaluated with the SPSS 29 program (IBM Corp., Armonk, NY).

## Results

A total of 60 MRI scans were included in the analysis, comprising 30 cases with confirmed biceps pulley lesions and 30 cases without lesions, as determined by arthroscopy as the gold standard. Five independent observers evaluated the scans for 6 diagnostic signs associated with biceps pulley lesions. The mean patient age was 58.4 ± 10.9 years old, with nearly one half being male patients (27/60; 45%). In addition, 67% of shoulders were right-sided.

The link between MRI signs and a correct diagnosis is shown in Table 1. Two of 5 assessors showed significant associations with correctly diagnosing a biceps pully lesion and identifying the edema and displacement signs. The rest of the authors did not show a statistically significant association with these signs. In contrast, only one assessor reported a significant link between correctly diagnosing biceps pulley lesion and identifying the angle, stamp, and distance signs.

**Table 1:** Precision of the Certainty Diagnosis of Biceps Pulley Lesions and the Presence of MRI Signs per Assessor

Assessor	MRI Signs					
	Stamp	Edema	LHTB Angle	LHBT Groove Distance	Instability	Displacement
1						
OR	2.73	3.5	2.8	ND	ND	ND
95% CI	0.6-11.8	0.6-18.9	0.4-15.7	ND	ND	ND
P value	.299	.254	.424	**.005**	**.024**	.237
2						
OR	9.75	7.5	0.8	ND	3.2	4.5
95% CI	2.7-35.1	2.24-25.0	0.6-0.9	ND	0.8-11.8	1.09-18.5
P value	**<.001**	**<.001**	**.024**	0.112	0.125	.057
3						
OR	2.19	1.3	14.5	ND	ND	5
95% CI	0.7-3.7	0.3-4.2	1.7-122.3	ND	ND	1.5-16.5
P value	.267	.718	**.006**	.237	**.024**	.13
4						
OR	8.1	6	4.2	ND	ND	6
95% CI	1.6-40.7	1.4-24.2	0.8-22.5	ND	ND	1.89-19.4
P value	**.01**	**.015**	.145	.112	.237	**.003**
5						
OR	2.14	2.66	7	5	ND	9.3
95% CI	0.6-7.3	0.8-4.6	1.3-35.4	0.9-17.3	ND	1.86-46.6
P value	.36	.091	**.021**	.08	**.002**	**.005**

NOTE. Assessors 1-3 are surgeons, whereas 4 and 5 are musculoskeletal radiologists.

CI, confidence interval; LHBT, long head biceps tendon; MRI, magnetic resonance imaging; ND, not determined. OR, odds ratio.

The overall diagnostic performance of MRI in detecting biceps pulley lesions is shown in Table 2, with a sensitivity of 81% and a specificity of 79%. Both the PPV and NPV were 80%. Among the individual signs, LHBT angle had the greatest sensitivity at 84%. In contrast, LHBT-groove distance exhibited the highest specificity at 98%.

**Table 2:** Diagnostic performance for Biceps Pulley Lesions overall and per MRI Sign

	Overall	MRI Signs					
		Stamp	Edema	LHTB Angle	LHBT Groove Distance	Instability	Displacement
Sensitivity	81%	29%	29%	21%	15%	19%	30%
Specificity	79%	81%	83%	94%	98%	96%	85%
PPV	80%	71%	72%	84%	93%	89%	78%
NPV	80%	41%	42%	43%	45%	44%	40%
Accuracy	50%	49%	50%	50%	50%	50%	50%

LHBT, long head biceps tendon; MRI, magnetic resonance imaging; NPV, negative predictive value; PPV, positive predictive value.

Interobserver reliability analysis, measured using Cohen’s kappa coefficients, revealed a moderate global kappa value of 0.59. Pairwise comparisons showed variable kappa values (Table 3). Notably, 2 assessor pairs (e.g., 2-4, and 3-4) consistently achieved greater agreement values compared with other pairs. When analyzing signs, the stamp and luxation signs had the highest and lowest agreement scores, respectively. When comparing radiologists with shoulder surgeons, radiologists had similar average metrics across all evaluated parameters: sensitivity (82% vs 79%), specificity (80% vs 79%), positive predictive value (80% vs 79%), and negative predictive value (82% vs 79%) (Table 4).

**Table 3:** Inter-rater Agreement, as Shown With Assessor Pairs, for the Diagnosis of Biceps Pulley Lesions and MRI Sign Identification

	Assessors									
	1∼2	1∼3	1∼4	1∼5	2∼3	2∼4	2∼5	3∼4	3∼5	4∼5
Overall, k	0.63	0.57	0.60	0.53	0.60	0.63	0.57	0.67	0.60	0.63
Stamp, k	0.63	0.56	0.60	0.53	0.60	0.63	0.56	0.66	0.60	0.63
Edema, k	0.56	0.50	0.53	0.46	0.53	0.56	0.50	0.60	0.53	0.56
LHBT angle, k	0.50	0.46	0.43	0.40	0.53	0.50	0.46	0.56	0.53	0.50
LHBT-groove distance, k	0.53	0.46	0.50	0.43	0.50	0.53	0.46	0.56	0.50	0.53
Instability, k	0.46	0.43	0.40	0.36	0.50	0.46	0.43	0.53	0.50	0.46
Displacement, k	0.50	0.46	0.43	0.40	0.53	0.50	0.46	0.56	0.53	0.50

NOTE. Assessors 1-3 are surgeons, whereas 4 and 5 are musculoskeletal radiologists.

k, Cohen's kappa value; LHBT, long head biceps tendon; MRI, magnetic resonance imaging;

**Table 4:** Comparison of Overall Diagnostic Performance Between Shoulder Surgeons and Musculoskeletal Radiologists for the Diagnosis of Biceps Pulley Lesions With MRI

	Shoulder Surgeons	Radiologists
	Assessors 1, 2, and 3	Assessors 4 and 5
Sensitivity	79%	82%
Specificity	79%	80%
PPV	79%	80%
NPV	79%	82%

MRI, magnetic resonance imaging; NPV, negative predictive value; PPV, positive predictive value.

## Discussion

Overall diagnostic performance metrics for MRI included a sensitivity of 81%, specificity of 79%, PPV of 80%, and NPV of 80%. LHBT angle showed the greatest sensitivity (84%) and LHBT-groove distance showed the greatest specificity (98%). Interobserver reliability analysis revealed moderate agreement overall, with a global kappa value of 0.59. Substantial agreement was achieved in certain observer pairs, though variability across signs highlighted the need for standardized diagnostic criteria and training to enhance consistency.

Biceps pulley lesions are a common cause of anterior shoulder pain, often occurring alongside associated shoulder pathologies.^2^^,^^3^^,^^6^^,^^12^ Braun et al.^2^ previously reported a prevalence of up to 32% in a large cohort of more than 200 patients with anterior shoulder pain and showed significant correlations with LHBT subluxation/dislocation, rotator cuff injuries, and SLAP lesions. Given the lack of specificity in clinical examination, imaging plays a critical role in diagnosis.^3^^,^^7^

Conventional MRI and MR arthrogram are the most frequently used imaging modalities, although their diagnostic performance has varied across studies.^3^^,^^7^^,^^8^^,^^13^ In their retrospective analysis of 28 MR arthrograms with 3 assessors, Schaeffeler et al.^14^ reported a sensitivity of 82% to 89% and a specificity of 87 to 98%. This aligns with Weishaupt et al.,^15^ who reported a sensitivity of 86% and 93% (assessors 1 and 2, respectively) and specificity of 100% and 80% for MR arthrography, in a smaller sample. By comparison, Ebrahimi Ardjomand et al.,^16^ in a study of 68 conventional MRIs, reported sensitivities of 95%, 88%, and 93% with specificities of 62%, 73%, and 81% among 3 assessors. In our study, global sensitivity and specificity were 81% and 79%, respectively. Interestingly, Nada et al.,^17^ analyzing 84 conventional MRIs, reported sensitivity between 66% and 78% and specificity of 90% to 92%. The variability in results across studies underscores the importance of standardized diagnostic criteria, such as MRI signs of LHBT instability. Moreover, the results from these studies show that conventional MRI is sufficient to diagnose biceps pulley lesions.

It is well known that chronic LHBT instability produces tendinopathy, which is one of the most commonly indicative signs.^16^^,^^18^^,^^19^ Schaeffeler et al.,^14^ Nada et al.,^17^ and Ebrahimi Ardjomand et al.^16^ reported tendinopathy as the most sensitive sign, followed closely by the displacement sign. Although the displacement sign was also the second most sensitive sign in our study, the LHBT angle emerged as the most sensitive. Specificity, in contrast, showed greater variability. Nada et al.^17^ and Ebrahimi Ardjomand et al.^16^ reported LHBT luxation as the most specific sign, a finding consistent with our results, where LHBT luxation was second only to LHBT-groove distance. This highlights the reliability of LHBT luxation and displacement as diagnostic indicators, as described by Zappia et al.,^10^ for the diagnosis of biceps pulley lesions.

Inter-rater agreement in our study was globally moderate, although agreement varied significantly depending on the sign as shown in other studies.^14^^,^^16^^,^^17^ For instance, Ebrahimi Ardjomand et al.^16^ reported a 0.47 to 0.74 Cohen’s kappa for the displacement sign in comparison to the fair-to-moderate Cohen’s kappa in our study (0.40-0.56). In contrast, although they reported fair agreement (Cohen’s kappa of 0.36) for LHBT luxation, we found moderate agreement in 8 occasions, with the greatest associations being between assessors 2-3 (Cohen’s kappa of 0.50), 3-4 (0.53), and 3-5 (0.50). However, their global inter-rater agreement was substantial (0.75) compared with the moderate agreement found in our study (0.59). It is important to note that in the study by Ebrahimi Ardjomand et al., authors included greater-quality MRIs (3 T) and all 3 assessors were radiologists. Despite variability in sign identification, global inter-rater agreement can be moderate to substantial for the diagnosis of pulley lesions. This variability in sign identification calls for a more standardized approach to diagnosing biceps pulley lesions.

Radiologists and surgeons had similar accuracy in our study. There are only a few studies comparing radiologists with surgeons for the detection of shoulder lesions through imaging.^20^^,^^21^ In a retrospective case-series of 1,090 patients, Kilic et al.^20^ compared radiologist versus surgeon accuracy for the detection of subscapularis tears through MRI. Although they reported greater surgeon precision, we found similar accuracy between radiologists and surgeons. However, the surgeon in their study had the benefit of a clinical examination in conjunction with an MRI, while in our study all assessors analyzed MRIs without a clinical examination and radiologists were experts in musculoskeletal radiology.

### Limitations

Several limitations must be acknowledged. First, the retrospective study design introduces inherent biases. Second, although a fellowship-trained shoulder surgeon performed arthroscopies, this procedure remains operator-dependent and subject to variability in the interpretation of shoulder pathologies. Third, the risk of imaging report exposure may have inadvertently influenced the focus on specific pathologies. Fourth, the sample size in this study was small, potentially limiting generalizability. Fifth, MRIs were obtained from different sites, possibly affecting reliability. Finally, the inclusion of only patients with both MRI and subsequent arthroscopy introduces the possibility of spectrum bias.

## Conclusions

Overall, conventional MRI had acceptable diagnostic performance, with sensitivity, specificity, and predictive values of approximately 80%. Among the evaluated signs, the LHBT angle had the greatest sensitivity, whereas the LHBT-groove distance showed the greatest specificity. Interobserver reliability was moderate overall, although some observer pairs achieved substantial agreement. However, there was variability across diagnostic signs.

## Disclosures

The authors declare the following financial interests/personal relationships which may be considered as potential competing interests: F.M. reports consulting or advisory with Arthrex and Exactech. A.L. reports consulting or advisory with Arthrex, Medacta International SA, Enovis Corporation, and Stryker. P.D. reports consulting or advisory, funding grants, speaking and lecture fees, and travel reimbursement from Arthrex. A.G-A. reports consulting or advisory with Arthrex and co-author and cofounder of BeeMed, The HIVE, Med4Cast, and FORE-AL and a shareholder of BeeMed. All other authors (J.A., R.E-V., E.V-R., A.L-V., G.F-d.R., J.C-L., J.P.M.) declare that they have no known competing financial interests or personal relationships that could have appeared to influence the work reported in this paper.

## References

[1] Lafosse L., Reiland Y., Baier G.P., Toussaint B., Jost B. (2007). Anterior and posterior instability of the long head of the biceps tendon in rotator cuff tears: A new classification based on arthroscopic observations. Arthroscopy.

[2] Braun S., Horan M.P., Elser F., Millett P.J. (2011). Lesions of the biceps pulley. Am J Sports Med.

[3] Martetschläger F., Zampeli F., Tauber M., Habermeyer P. (2020). Lesions of the biceps pulley: A prospective study and classification update. JSES Int.

[4] Nakata W., Katou S., Fujita A., Nakata M., Lefor A.T., Sugimoto H. (2020). Biceps pulley: Normal anatomy and associated lesions at MR arthrography. RadioGraphics.

[5] Tang X., Zhang J., Zhang J., He Y. (2023). Correlation between the morphological features of the biceps groove and injuries to the biceps pulley and the long head tendon of the biceps. BMC Musculoskelet Disord.

[6] Baumann B., Genning K., Böhm D., Rolf O., Gohlke F. (2008). Arthroscopic prevalence of pulley lesions in 1007 consecutive patients. J Shoulder Elbow Surg.

[7] Kang Y., Lee J.W., Ahn J.M., Lee E., Kang H.S. (2017). Instability of the long head of the biceps tendon in patients with rotator cuff tear: Evaluation on magnetic resonance arthrography of the shoulder with arthroscopic correlation. Skeletal Radiol.

[8] Diplock B., Hing W., Marks D. (2023). The long head of biceps at the shoulder: A scoping review. BMC Musculoskelet Disord.

[9] Tarallo N., C, Morgano M., Curti M., Spanò E., Castagna A., Genovese E.A. (2021). Intra-articular long head of the biceps tendon: Magnetic resonance-arthrography classification and review of literature. Pol J Radiol.

[10] Zappia M., Ascione F., Di Pietto F. (2021). Long head biceps tendon instability: Diagnostic performance of known and new MRI diagnostic signs. Skeletal Radiol.

[11] Landis J.R., Koch G.G. (1977). The measurement of observer agreement for categorical data. Biometrics.

[12] Gaskill T.R., Braun S., Millett P.J. (2011). The rotator interval: Pathology and management. Arthroscopy.

[13] Fischetti M., Stoppino L.P., Petrera M.R. (2022). MRI morphological evaluation of humeral head bone profile inside region of the biceps pulley reflection. Skeletal Radiol.

[14] Schaeffeler C., Waldt S., Holzapfel K. (2012). Lesions of the biceps pulley: Diagnostic accuracy of MR arthrography of the shoulder and evaluation of previously described and new diagnostic signs. Radiology.

[15] Weishaupt D., Zanetti M., Tanner A., Gerber C., Hodler J. (1999). Lesions of the reflection pulley of the long biceps tendon. Invest Radiol.

[16] Ebrahimi Ardjomand S., Meurer F., Ehmann Y. (2024). Evaluation of conventional MR imaging of the shoulder in the diagnosis of lesions of the biceps pulley. Acad Radiol.

[17] Nada M.G., Almalki Y.E., Basha M.A.A. (2024). Biceps pulley lesions: Diagnostic accuracy of nonarthrographic shoulder MRI and the value of various diagnostic signs. J Magn Reson Imaging.

[18] Werner A., Ilg A., Schmitz H., Golhke F. (2003). Tendinitis of the long head of biceps tendon associated with lesions of the “biceps reflection pulley”. Sportverletzung · Sportschaden.

[19] Nho S.J., Strauss E.J., Lenart B.A. (2010). Long head of the biceps tendinopathy: Diagnosis and management. J Am Acad Orthop Surg.

[20] Kilic A.I., Ardebol J., Ghayyad K., Pak T., Menendez M.E., Denard P.J. (2024). Both radiologists and surgeons miss a substantial number of subscapularis tears on magnetic resonance imaging examination prior to shoulder arthroscopy. Arthrosc Sports Med Rehabil.

[21] Stanborough R.O., Garner H.W., Simovitch R.W., Schoch B.S. (2024). Magnetic resonance imaging of the shoulder: Interpretation of common orthopaedic injuries. J Am Acad Orthop Surg.

